# GpABC: a Julia package for approximate Bayesian computation with Gaussian process emulation

**DOI:** 10.1093/bioinformatics/btaa078

**Published:** 2020-02-05

**Authors:** Evgeny Tankhilevich, Jonathan Ish-Horowicz, Tara Hameed, Elisabeth Roesch, Istvan Kleijn, Michael P H Stumpf, Fei He

**Affiliations:** b1 Centre for Integrative Systems Biology and Bioinformatics, Department of Life Sciences, Imperial College London, London SW7 2AZ, UK; b2 Melbourne Integrative Genomics, University of Melbourne, Parkville, VIC 3010, Australia; b3 School of Computing, Electronics, and Mathematics, Coventry University, Coventry CV1 2JH, UK

## Abstract

**Motivation:**

Approximate Bayesian computation (ABC) is an important framework within which to infer the structure and parameters of a systems biology model. It is especially suitable for biological systems with stochastic and nonlinear dynamics, for which the likelihood functions are intractable. However, the associated computational cost often limits ABC to models that are relatively quick to simulate in practice.

**Results:**

We here present a Julia package, GpABC, that implements parameter inference and model selection for deterministic or stochastic models using (i) standard rejection ABC or sequential Monte Carlo ABC or (ii) ABC with Gaussian process emulation. The latter significantly reduces the computational cost.

**Availability and implementation:**

https://github.com/tanhevg/GpABC.jl.

## 1 Introduction

Parameter estimation and model selection are the central tasks in reverse engineering of cellular systems. Although identifying the best parameter value or model structure is of obvious interest, so too is the evaluation of the estimation uncertainty under the Bayesian framework. Much of the work has focused on Approximate Bayesian Computation (ABC) for both parameter and model inference (Toni *et al.*, 2009), since the likelihood of nonlinear biological models is often intractable. A number of improvements on the basic ABC rejection scheme have been proposed, employing either Markov Chain Monte Carlo (ABC-MCMC) (Marjoram *et al.*, 2003) or sequential Monte Carlo (ABC-SMC) (Sisson *et al.*, 2007; Toni *et al.*, 2009). However, even with these optimizations, the number of time-consuming model simulations required can still easily reach the millions. A further speed-up of ABC can be achieved by employing emulation techniques, where a mapping between the parameters and the approximated likelihood of a complex model (i.e. discrepancy between the model outputs and measurement data) is built using statistical regression models. Only a small number of simulations is required to train the emulator, which can then be used to predict the model outputs (or the discrepancy) for other parameters with a significantly lower computational cost. Accelerating ABC or MCMC with emulation has been proposed using either local regression or Gaussian process (GP) (Jabot *et al.*, 2014; Meeds and Welling, 2014; Wilkinson, 2014). The GP-based approach has gained more traction recently, due to its inherent ability to quantify uncertainty and increased availability of computational resources.

A number of ABC packages have been published (for a review, see Kousathanas *et al.*, 2018) including a Julia package ApproxBayes.jl; however, a package with a focus on emulation is still lacking. Here, we present a Julia package, GpABC, which implements rejection ABC and ABC-SMC with GP emulation for parameter and model inference in deterministic and stochastic models. Details of the algorithms, documentation and examples are available online. An alternative approach to modeling the joint density of the parameters and the discrepancy with a GP has been proposed in the Approximate ABC (AABC) algorithm (Buzbas and Rosenberg, 2015). AABC models the joint density of the data and parameters as a mixture of a relatively small number of model simulations.

## 2 Materials and methods

### 2.1 ABC and GP emulation

The simplest version of ABC, ABC rejection, proceeds as follows: (i) sample a parameter vector, or particle, *θ* from the prior distribution; (ii) simulate a dataset *D* from the model given *θ* and compute summary statistics S(D|θ); (iii) compute a distance *d* that quantifies the discrepancy between S(D|θ) and the statistics of observed data $S(D^{*})$; and (iv) accept *θ*, if the distance $d(S(D|\theta ),S(D^{*}))$ is less than some threshold value *ε*. This process is repeated multiple times to obtain the approximated posterior distribution. ABC-SMC algorithm (Toni *et al.*, 2009) speeds up the rejection ABC by constructing a set of intermediate distributions, which are defined by a sequence of threshold values $\varepsilon_{t}$ in decreasing order, $\varepsilon_{1}>\varepsilon_{2}>\ldots >\varepsilon_{T}\geq 0$. Each intermediate distribution is generated from the previous using a sequential importance sampling scheme.

To reduce the computational cost of running a large number of simulations in ABC, a GP emulator is first constructed to quantify the mapping from model parameter *θ* to the aforementioned distance *d*: $d(S(D|\theta ),S(D^{*}))\equiv d(\theta )∼GP(\mu (\theta ),k(\theta ,\theta \prime ))$.

**Fig. 1.**
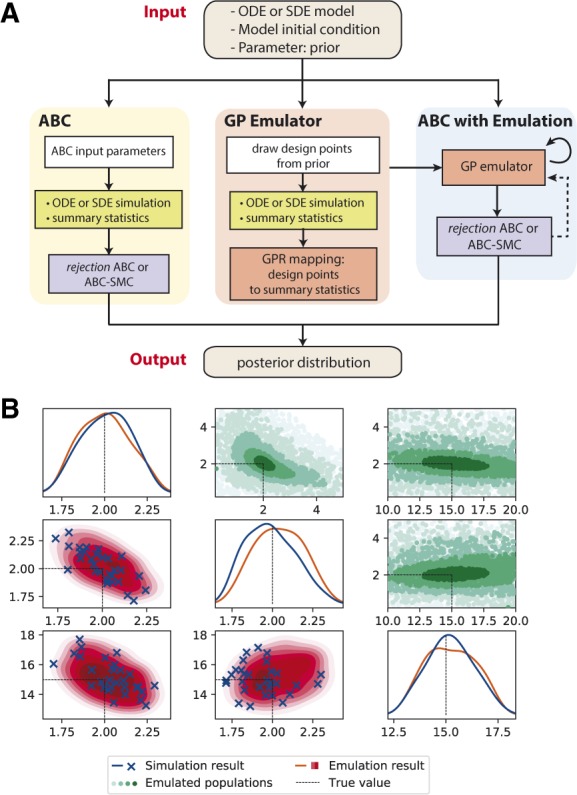
(**A**) Schematic diagram of GpABC package structure. The software can perform either purely Monte Carlo simulation-based ABC (i.e. rejection ABC or ABC-SMC) or computational efficient ABC with emulation, where the GP emulator is first (re-)trained based on simulation from selected design points in the prior. Dashed arrow indicates the emulator re-training can be part of the ABC-SMC algorithm as design points are selected from different SMC populations iteratively. (**B**) Parameter inference results of a three-parameter deterministic model using ABC-SMC simulation and emulation. Subplots on the diagonal and lower triangular show marginal and joint posterior distributions of parameter estimates in the final ABC-SMC population (simulation in blue and emulation in red). Scatterplots above the diagonal show ABC-SMC populations with GP emulations. (Color version of this figure is available at *Bioinformatics* online.)

This GP is (re-)trained based on simulations of a relatively small number of parameters $\theta ={\left[\theta_{1},\ldots ,\theta_{n}\right]}^{T}$, referred to as design points, from the prior or the posterior of the previous step in ABC-SMC, and selected in a way to control both the emulation accuracy and computational efficiency. Prediction of the distance for other particles can then be obtained from the GP posterior, without simulating the model. The GP emulation and re-training process can then be used with either rejection ABC or ABC-SMC algorithms (Fig. 1).

### 2.2 Stochastic simulation and model selection

Biochemical reactions are stochastic in nature, and the distribution of stochastic simulation trajectories is generally non-Gaussian. To meet the Gaussian noise assumption of a GP and to consider computational efficiency, we employ the linear noise approximation for stochastic simulation. Users can select whether to perform ABC or ABC emulation for deterministic or stochastic modeling. In addition to parameter inference, a model selection algorithm (Toni *et al.*, 2009) is also implemented, where model indicators *m* are treated as an extra parameter. The joint posterior distribution over the combined space of models and parameters p(m,θ|D) can be obtained via an ABC-SMC scheme, and finally, the p(m|D) is obtained by marginalizing over parameters.

### 2.3 Package overview and features

Users can easily choose or define several parameters of the algorithm. For ABC, these are the summary statistics S(D|θ) or a subset of the model’s outputs, prior distributions, distance function *d*, the number of accepted parameters in the posterior and threshold values $\varepsilon_{t}$. The latter strongly depend on the dynamics of the biochemical process and the noise level. Users can also choose how to select the design points in the emulator re-training process (with several optional strategies).

Package outputs include posterior populations of accepted parameters for each threshold value, as well as distances, *d*, for each accepted parameter. Whenever emulation is used, additional information about the GP emulator is also provided. Performance depends on how well the model fits the data and the choice of thresholds *ε*. In simulation mode, the model is simulated on each attempt, so the cost of simulating the model has crucial impact on performance. In emulation mode, the model is simulated only for training the emulator; subsequently model emulation is done in batches. Training the GP has computational complexity $O(n^{3})$, and emulating the model has complexity of O(bn), with batch size *b*.

In summary, GpABC is a user-friendly and extendable Julia package that can perform simulation-based ABC or ABC with GP emulation, for both deterministic and stochastic systems biology models. The package can be used to infer the posterior parameter distribution or select the best model structure that represents the data from candidate models.

## Funding

This work was supported by Biotechnology and Biological Sciences Research Council [BB/N003608/1] and by Wellcome Trust PhD awards to J.I.-H., T.H. and I.K [108908/B/15/Z, 215358/Z/19/Z, 215359/Z/19/Z, 203968/Z/16/Z].

*Conflict of Interest*: none declared.
